# Impact of Erbium and Gadolinium on *Xenopus laevis* Embryo Development: A Study of Rare Earth Element Toxicity

**DOI:** 10.3390/ijms27072920

**Published:** 2026-03-24

**Authors:** Chiara Fogliano, Simona Di Marino, Giulia Rusciano, Francesca Vignola, Adriana Petito, Paola Venditti, Patrizia Cretì, Chiara Maria Motta, Bice Avallone, Rosaria Scudiero, Rosa Carotenuto

**Affiliations:** 1 Department of Biology, University of Naples Federico II, 80126 Naples, Italy; chiara.fogliano@unina.it (C.F.); simona.dimarino@unina.it (S.D.M.); adriana.petito@unina.it (A.P.); paola.venditti@unina.it (P.V.); chiaramaria.motta@unina.it (C.M.M.); bice.avallone@unina.it (B.A.); 2 Department of Physics “Ettore Pancini”, University of Naples Federico II, 80126 Naples, Italy; giulia.rusciano@unina.it; 3 Department of Biological and Environmental Sciences and Technology, University of Salento, 73100 Lecce, Italy; patrizia.creti@unisalento.it

**Keywords:** REEs, embryonic development, oxidative stress, gene expression, Raman spectroscopy

## Abstract

Rare earth elements (REE), such as gadolinium (Gd) and erbium (Er), are increasingly recognised as emerging environmental contaminants due to their widespread use in industrial processes, electronics, and medical imaging applications. Despite their extensive presence in aquatic ecosystems, little is known about their developmental toxicity. In this study, *Xenopus laevis* embryos were exposed to environmentally relevant concentrations of Gd and Er during critical early developmental stages. The assessed endpoints included survival, malformations, growth (body length), and heart rate. Both Gd and Er caused significant sublethal effects, including increased axial malformations, reduced growth, and altered cardiac activity. To explore potential mechanisms of toxicity, the expression patterns of key developmental genes (*fgf8*, *bmp4*, *sox9*, *egr2*, *rax1*, *pax6*) and pro-inflammatory cytokines (*tnfα*, *il1β*, *p65*) were analysed using Real-Time PCR. The results showed dysregulation of gene expression, indicating disruption to pathways involved in morphogenesis and neurodevelopment. Elevated reactive oxygen species levels suggested that oxidative stress was a contributing factor. Raman spectroscopy confirmed biochemical changes affecting proteins, lipids, and nucleic acids, providing evidence of cellular stress and metabolic imbalance. Overall, our findings demonstrate that even low-level exposure to Gd and Er can impair amphibian embryonic development and disturb molecular homeostasis. These results emphasise the ecological risks of REE pollution and highlight the importance of ongoing environmental monitoring and long-term toxicological research.

## 1. Introduction

Rare earth elements (REEs) include 15 lanthanides, scandium, and yttrium [1]. Classified as either light or heavy [2], they exhibit distinctive magnetic, luminescent, and electrochemical properties [3], enabling a wide range of high-tech applications, including in electronics, renewable energy technologies, and industrial processes [4]. REEs have also found applications as tracers in fields such as agriculture, geochemistry, and environmental chemistry [5,6].

As the global demand for advanced technologies increased, REEs became increasingly important to emerging sectors [7], raising concerns about the environmental risks associated with their release into natural ecosystems [8,9,10].

In aquatic systems, sediments are a primary reservoir for REEs, allowing these elements to persist and potentially bioaccumulate within the food web [11]. Significant ecological and health risks to humans, plants, and aquatic organisms have been demonstrated [12]. Nevertheless, few studies have addressed long-term toxicity, particularly in freshwater animals [13].

Among the lanthanides, gadolinium (Gd) has become a notable aquatic pollutant, mainly due to its widespread use in medical diagnostics as a contrast agent [14] and its growing application in industrial and domestic sectors [15]. Elevated Gd levels, up to 80 µg/L, have been recorded near sewage treatment plants [6] and in other environmental matrices, since traditional purification methods only remove around 10% of Gd from wastewater [16]. Similarly, significant erbium (Er) contamination has been detected in water [17].

REEs exposure causes oxidative stress and widespread cell damage [18,19], and disrupts mitosis [20,21]. Based on this evidence, the study aimed to evaluate the effects of Gd and Er exposure on a rapidly dividing model: the *Xenopus laevis* embryo. This species is a well-established model in ecotoxicology due to the transparency of its embryos and their biological relevance to vertebrates, including humans [22].

The investigation employed the standard Frog Embryo Teratogenesis Assay-*Xenopus* (FETAX) test, focusing on key endpoints such as survival, malformations, growth, and heart rate. To clarify the molecular mechanisms behind developmental disruption, the expression of the early embryonic developmental genes *fgf8*, *bmp4*, *sox9*, *egr2*, *rax1*, and *pax6* was analysed. The potential immunological and detoxification responses to exposure were also assessed by measuring the expression of pro-inflammatory cytokines (*tnfα*, *il1β*, and *p65*) and detoxification-related genes (*abcb1*). Raman spectroscopy identified compositional differences, while quantification of reactive oxygen species (ROS) was used to assess the oxidative state. Combining morphological, molecular, and biochemical analyses will provide a thorough understanding of how these two REEs impact early development at both phenotypic and molecular levels. Exposure at environmentally relevant levels will yield valuable insights into their potential ecotoxicological effects.

## 2. Results

### 2.1. Effects of Er and Gd on Embryo Survival and Growth

**Figure 1:**
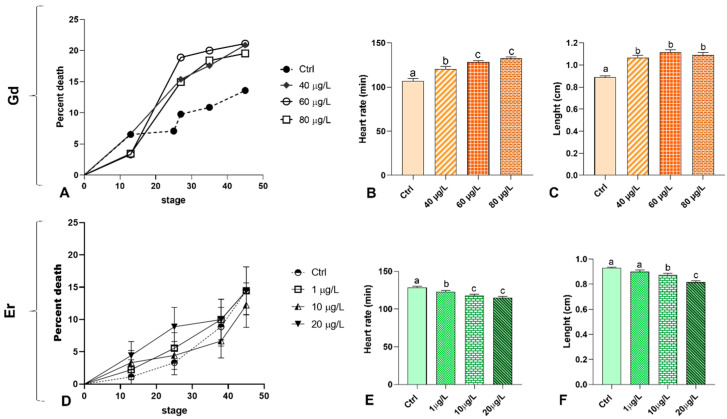
Mortality (%), heart rate (beats/min) and growth retardation in *Xenopus laevis* embryos exposed to gadolinium (Gd) (**A**–**C**) or erbium (Er) (**D**–**F**) (*n* = 3 replicate experiments; 90 embryos/treatment). Different letters (a–c) indicate statistically significant differences among groups (*p* < 0.05; One-way ANOVA). Groups sharing the same letter are not significantly different.

Following treatment, there was no significant increase in mortality compared to the control group. Gd treatment resulted in a peak mortality rate of just over 20%, with no clear dose-dependent trend. In controls, the maximum rate was 13% (Figure 1A). After Er treatment, the mortality rate was about 13% in the control and all treated groups (Figure 1D). The two treatments exhibited distinct effects on heart rate. Gd induced a significant, dose-dependent tachycardia (Figure 1B), whereas the Er caused dose-dependent bradycardia (Figure 1E). The treatments also had opposite effects on embryonic growth. Gd-treated embryos were significantly longer than the controls (Figure 1C), whereas Er-treated embryos were smaller, but only at the two highest doses (Figure 1F).

### 2.2. Effects of Gd and Er on Embryo Phenotype

**Figure 2:**
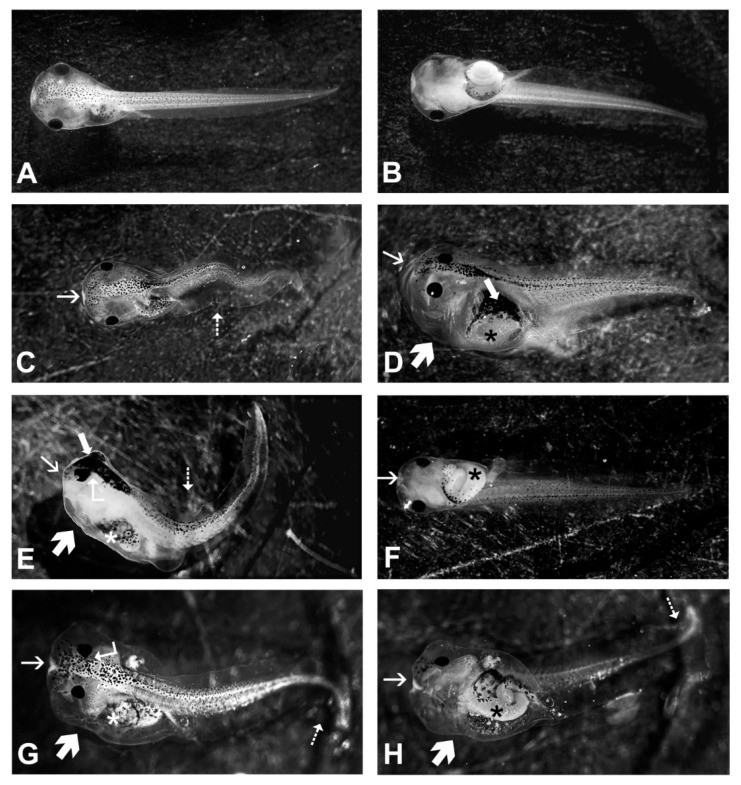
Representative images of morphological alterations observed in *Xenopus laevis* embryos exposed to gadolinium (Gd) (**C**–**G**) or erbium (Er) (**D**–**H**) relative to control embryos (**A**,**B**). Thin arrow: head deformities; arrow: altered pigmentation; thick arrow: oedema; dotted arrow: trunk bending; asterisk: gut abnormalities; bent arrow: eye deformities.

Both treatments resulted in a low malformation rate. Gd exposure led to a slight increase in phenotypic anomalies, which reached a peak of 8.4%, with no clear dose-dependent pattern (Table 1). By contrast, Er caused a dose-dependent increase in the malformation rate, reaching statistical significance at concentrations of 10 μg/L and 20 μg/L, with rates of 7.9% and 11.5%, respectively (Table 1). The types of anomalies were similar across both treatments but were not correlated with dose. These mainly included head and abdominal oedema, head deformities (sometimes heart-shaped), trunk bending, variations in eye size and shape (appearing either enlarged or reduced) and altered pigmentation distribution (Figure 2).

### 2.3. Effects of Gadolinium and Erbium on Gene Expression

**Figure 3:**
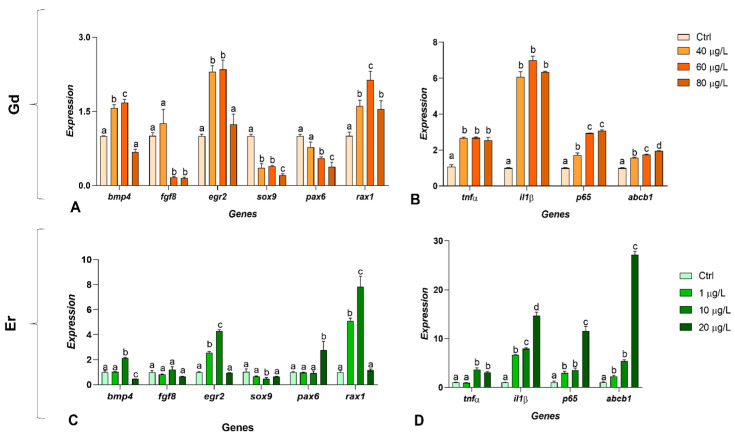
Real-Time PCR of *Xenopus laevis* embryos exposed to gadolinium (Gd) and erbium (Er). (**A**,**C**) Regulatory genes of early embryonic development. (**B**,**D**) Genes involved in inflammatory responses and multidrug resistance. Two-Way ANOVA with the Bonferroni post hoc test. Data are expressed as mean ± SD; different letters (a–d) indicate statistically significant differences among groups (*p* < 0.05). Groups sharing the same letter are not significantly different.

The gene expression analysis focused on key genes involved in early embryonic development and inflammatory responses. Concerning Gd, all genes related to early embryonic development were dysregulated, with no discernible dose-dependent trend. Specifically, *egr2*, and *rax1* were strongly upregulated, while *fgf8*, *sox9*, and *pax6* were downregulated; *bmp4* showed both upregulation (40 and 60 µg/L) and downregulation (80 µg/L) (Figure 3A). Gd caused a widespread and significant increase in the expression of inflammation-related genes. For the *abcb1* gene, the upregulation was dose-dependent (Figure 3B).

After exposure to Er, the situation was different: *egr2* and *rax1* were upregulated at concentrations of 1 μg/L and 10 μg/L, while *pax6* was only upregulated at the highest concentration. *bmp4* was upregulated at 10 μg/L and significantly downregulated at 20 μg/L (Figure 3C). Similarly, Er treatment caused a general and significant upregulation of inflammation-related genes and *abcb1* (Figure 3D).

A summary of the statistical values for both Gd and Er effects is provided in Supplementary Tables S1 and S2.

### 2.4. ROS Content Determination

**Figure 4:**
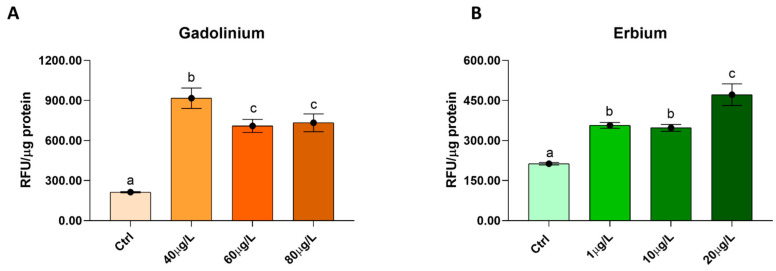
Effect of Gd (**A**) and Er (**B**) on ROS production in *Xenopus laevis* embryos. Data, expressed as the Relative Fluorescent Units (RFU) per microgram of protein, are means ± SDs of two independent measures. *n* = 3. Different letters (a–c) indicate statistically significant differences among groups (*p* < 0.05; One-way ANOVA). Groups sharing the same letter are not significantly different.

Total reactive oxygen species (ROS) increased significantly following exposure to both Gd (Figure 4A) and Er (Figure 4B). Specifically, for Gd, the increase was not dose-dependent, showing no proportional rise at higher concentrations. In contrast, treatment with Er showed an opposite pattern: ROS levels increased progressively with concentration, reaching the highest values at the highest tested dose although the increase was not strictly linear across all concentrations.

### 2.5. Raman Spectroscopy

**Figure 5:**
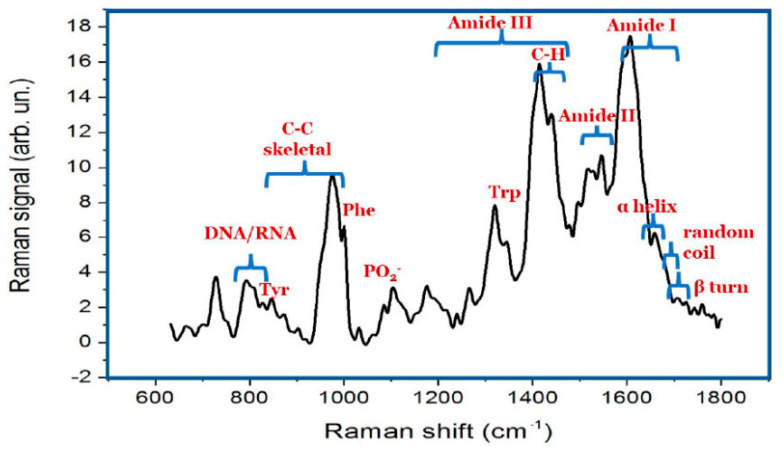
Mean spectrum (average of 10 signals) corresponding to the control sample. Labels indicate the main band assignment.

**Figure 6:**
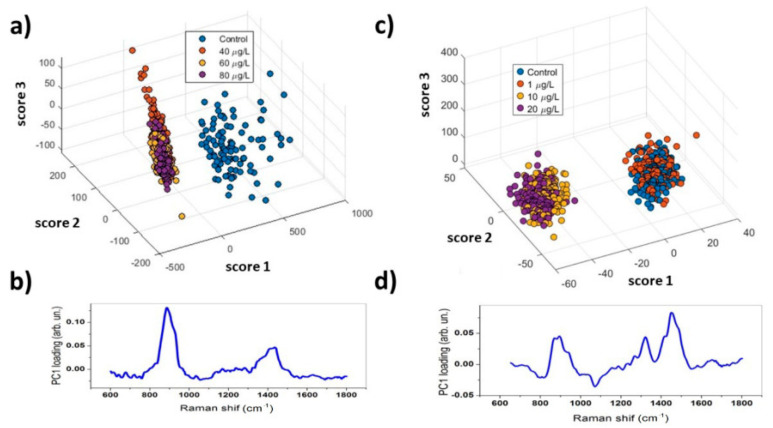
Outcomes of PCA performed on treated samples. Score plot (**a**) and PC1 loading plot (**b**) obtained for Gd-treated samples. Score plot (**c**) and PC1 loading plot (**d**) obtained for Er-treated samples.

As a preliminary step, Raman spectra of the control samples were acquired in the 500–1700 cm^−1^ range (Figure 5). The mean spectrum revealed characteristic bands attributable to proteins (Amide I at ~1640 cm^−1^, Amide II at ~1550 cm^−1^, Amide III at ~1300 cm^−1^), amino acids (e.g., phenylalanine at ~1001/1031 cm^−1^, tyrosine at ~830/850 cm^−1^, tryptophan at ~1340/1360 cm^−1^), lipids/phospholipids (~930, 1087, and 1400 cm^−1^), and nucleic acids (~780 and 1050 cm^−1^). To assess the chemical differences among the embryo extracts, 100 spectra were collected per sample and analysed using Principal Component Analysis (PCA). In Gd-treated samples (Figure 6a), PCA revealed a clear separation along PC1, with the treated samples showing lower scores. The corresponding loading plot showed reduced intensity of the lipid/protein-associated band at ~930 cm^−1^ and of the lipid band at ~1400 cm^−1^, suggesting a treatment-related degradation of lipid structures. Similarly, Er-treated samples (Figure 6b) showed differentiation based on PC1. Samples with higher Er concentrations clustered in the negative PC1 region, whereas controls and those with lower concentrations clustered in the positive PC1 region. Loadings revealed positive lipid-associated features at ~930 and 1400 cm^−1^, supporting the occurrence of lipid peroxidation. Additional changes included a broad peak around ~1650 cm^−1^ (potential protein denaturation), negative contributions at ~1080 cm^−1^ (DNA/RNA and phospholipids), and a positive signal at ~1320 cm^−1^, which also indicates biochemical perturbations following Er exposure.

## 3. Discussion

REEs are rare in Earth’s crust, and organisms have no protection against increases in their concentration caused by anthropogenic activities. Particular threats are posed to amphibians living in freshwater environments, which are, on average, highly polluted. They are highly vulnerable because they are exposed to deposition and have no protection beyond a jelly coat. Early exposure is associated with disturbances in the expression of master developmental genes that set the body plan and position, and regulate tissue differentiation.

Results obtained in this study indicate that neither Gd nor Er at the tested environmental concentrations increased mortality. In both cases, percentages remained close to or below the accepted 12%, which is considered physiological in FETAX tests [23,24]. The moderate increase observed after Gd exposure may be explained by the inhibition of esterase-β activity, as was reported in Chironomid larvae, leading to alterations in detoxification and metabolic processing pathways [25]. In any case, it must be noted that the analysis ended at stage 45/46; therefore, it can be assumed that mortality increased in later stages, as suggested by the several anatomical and physiological defects observed in the embryos.

The first evidence of stress is the dose-dependent change in heart rate. Interestingly, Gd and Er produced opposite effects: Gd induced tachycardia, whereas Er caused bradycardia. The depressant effect of Er has already been reported in mammals [26], whereas for Gd, both no effect [27] and increased heart rate [28] have been reported. Lanthanides have complex effects on cardiac Na channels. Gd, for example, inhibits Na currents but can also exert opposite, paradoxical effects [29]. In addition, if interference is exerted during organogenesis, it may cause significant impairments in adults. Consequences for ion channel function [30], including stretch-activated ion channels [31], have been reported.

The mechanisms underlying the observed heart responses may also include differences in the onset of sympathetic and parasympathetic cardiac tones [26] and effects on the central nervous system [32]. Not to be neglected is the correlation between oedema and rate anomalies. Malfunction of cardiac mechanosensors [33] is associated with cardiac dysfunction [34] and developmental defects [35]. In our model, the observed dysregulation of *bmp4* suggests an improper heart tube looping [36].

Heart rate and embryo lengthening are generally positively correlated, although this relationship may vary with developmental stage and metabolism [37]. In our *Xenopus* embryos, Gd promoted cell proliferation [38], resulting in increased length and heart rate. Conversely, Er, which has no significant proliferative effects [39], primarily acts as a pro-apoptotic factor [40,41], thereby decreasing heart rate and slowing growth.

REE-induced stress is also evidenced by increased ROS production. A pronounced dose dependence is observed after Er exposure, but higher prooxidant effects are observed with Gd. Raman spectroscopy confirms the evidence by clearly distinguishing controls from all exposed embryos. Marked prooxidant activity of Gd and Er has been demonstrated in many aquatic organisms [9,19,39]. For both REEs, increased ROS and NO production were reported, along with significant lipid peroxidation.

As expected, the increase in ROS is accompanied by a parallel increase in the malformation rate [42,43,44]. Teratogenicity of both Er and Gd has been associated with dysregulation of mitosis [18] and apoptosis [40], as well as with the onset of mitotic aberrations [39]. The rates increase in a dose-dependent manner in embryos exposed to Er, in line with the dose-dependent increase in ROS. In contrast, in embryos exposed to Gd, though peroxidation was consistent, malformation rates remained low at all concentrations. At present, it is unclear why. Gd is generally considered more toxic than Er [45], and the observed downregulation of *fgf8* and slight upregulation of the *abcb1* cassette in treated embryos suggest significantly impaired survival.

Regarding the type of malformation, the ones that are commonly reported in *Xenopus laevis* following exposure to various environmental contaminants [42,46,47] were observed. They can all be explained by the changes in expression of early developmental genes.

A common defect observed is gut malrotation. By disrupting cell rearrangement, differentiation, and proliferation, the two REEs caused insufficient gut lengthening, thereby reorienting intestinal rotation [48]. This alteration is typically accompanied by varying degrees of mucosal damage; therefore, the interference of the two REEs in reserve resorption should be evaluated. Instead, no interference with food resorption is expected since embryos at stage 45/46 do not feed. However, later interferences cannot be excluded.

Another common alteration observed is oedema. This primary sign of inflammation is associated with heart defects [49], which, in *Xenopus* embryos, are confirmed by dysregulation of the *bmp4* gene [36]. The upregulation of *il1β* and *tnfα* genes confirms the presence of an inflammatory response [41].

As expected, the embryos reacted to the REE insult by activating protective mechanisms. In addition to activating an antioxidant defence [19], the embryos overexpressed abcb1, a multixenobiotic resistance transporter, thereby protecting developing embryos from a plethora of toxicants, including tattoo ink [50], benzodiazepines [42], and microplastics [46], to name a few. Similarly, abcb1 activation occurs in zebrafish [51] or in sea urchin [52] embryos. Expression would begin at the morula stage [53], thereby providing an efficient protective mechanism during the activation of the master developmental gene. Exposed embryos also overexpress p65, a key subunit of the NF-κB transcription factor complex that activates target genes involved in axial patterning [54]. This dysregulation may explain the developmental defects observed [55], and, by triggering inflammation, the marked increase in il1β [56]. The upregulation of both p65 and abcb1 is particularly pronounced in Er-treated embryos, supporting previous studies indicating that Er is less toxic than Gd [45].

## 4. Materials and Methods

### 4.1. Animals Maintenance

Adult *Xenopus laevis* specimens, sourced from Nasco (Fort Atkinson, WI, USA), were maintained at the University of Naples Federico II in accordance with institutional and international animal welfare guidelines, including the NIH Guide for the Care and Use of Laboratory Animals and Italian (DL 116/92) and European regulations. Procedures minimised suffering and adhered to ministerial authorisation (Permit Number: 2013/0032839). Females and males were injected with 300 and 150 units of Gonase (IBSA Srl, Lodi, Italy), respectively, diluted in amphibian Ringer solution, to induce ovulation and spermatogenesis [46]. Fertilised eggs and embryos were obtained via in vivo insemination methods and staged according to Nieuwkoop and Faber [57].

### 4.2. Experimental Design: Exposure to Er and Gd

A total of three in vivo fertilisations were conducted using three different adult *Xenopus laevis* pairs for both the Gd and Er treatments. For the FETAX assay, embryos were exposed at the 4/8-cell stage in FETAX medium (106 mM NaCl, 11 mM NaHCO_3_, 4 mM KCl, 1 mM CaCl_2_, 4 mM CaSO_4_, and 3 mM MgSO_4_; 23), mimicking natural conditions [22,42,58].

At each fertilisation, 240 normally cleaved embryos were selected. These were placed in groups of 10, in triplicate, in 10-cm glass Petri dishes with 50 mL of FETAX (control) or solutions of Er (1, 10, and 20 μg/L) or Gd (40, 60, and 80 μg/L). Embryos were maintained until stages 45/46, with dead specimens removed daily. The experiments were carried out at 21 °C under a 12-h light/dark cycle, with daily pH checks (pH 7.4). Each test was repeated three times for the control and different compound concentration groups.

### 4.3. Heartbeat Rate Determination and Phenotype Analysis

The analyses were conducted at stage 45/46 (5 days post fertilisation). A total of 15 embryos from each treatment were randomly selected and placed under a stereomicroscope equipped with a video camera. Heart rate was determined from videos by counting the number of beats in three series of 30-s examinations carried out at 1-min intervals [42].

To determine the embryo’s length and possible phenotypic alterations, the embryos were anaesthetised in FETAX medium containing 100 mg/L MS-222 (SIGMA) and placed under an MZ16F UV stereomicroscope equipped with a Leica DFC 300Fx camera (Leica, Wetzlar, Germany). Length was determined with an eyepiece micrometre. Pictures taken both ventrally and dorsally were used to document the type and extent of the malformations. After analysis, they were processed for microscopy.

### 4.4. Real-Time PCR

To extract the total RNA, 15 embryos were selected from each treatment, and the Direct-zol RNA Mini Prep kit (ZymoResearch, Irvine, CA, USA) was used. RNA quality was assessed by measuring the 260/280 ratio spectrophotometrically. RNA was used for cDNA synthesis with the SuperScript Vilo cDNA Synthesis Kit (Life Technologies, Waltham, MA, USA). For each gene of interest, specific primer pairs were used [41] and tested in samples using PCR. Real-time PCR was performed with Power SYBR Green Master Mix (Life Technologies) using a 96-well optical reaction plate in a 20 µL total reaction volume. Reactions were conducted on an AriaMx Real-time PCR System. For relative transcript quantification, samples were normalised to ornithine decarboxylase (*odc*), a housekeeping control gene that was used to account for possible differences in the quantity and quality of the cDNAs used in the experiments. The magnitude of change in gene expression relative to the control was determined using the 2^−ΔΔCt^ method of Livak and Schmittgen [59].

### 4.5. Redox State Analysis

ROS production was assessed by measuring the conversion of 2′,7′-dichlorodihydrofluorescin diacetate (DCFH-DA) into the fluorescent compound dichlorofluorescein (DCF) through ROS-mediated processes [42]. Briefly, homogenates (25 µg of protein) from 15 randomly selected embryos per treatment were combined with 200 μL of a 0.1 M monobasic phosphate buffer at pH 7.4 containing 10 μM DCFH-DA. After a 15-min incubation, 100 μM FeCl3 was added, and the mixture was incubated for a further 30 min. The conversion of DCFH-DA into the fluorescent product DCF was measured using excitation and emission wavelengths of 485 nm and 530 nm, respectively. To assess background levels, the conversion of DCFH to DCF was also measured in the absence of homogenate. The results are expressed as Relative Fluorescent Units (RFU) per microgram of protein (µg protein^−1^).

### 4.6. Principal Component Analysis of Raman Spectra

Raman analysis of treated and untreated samples (15 randomly selected embryos/treatment) was performed using Principal Component Analysis (PCA), a well-known multivariate statistical tool for analysing multidimensional datasets. In this experiment, Raman spectra were pre-treated using a custom-made routine developed to eliminate spurious contributions from cosmic rays and to subtract a fourth-order polynomial background [60].

### 4.7. Statistical Analysis

Data were processed with GraphPad-Prism 8 software (GraphPad Software, Inc., San Diego, CA, USA). Survival distributions were assessed for significance using the Mantel–Cox test. To evaluate differences in heart rate, length, and oxidative stress, data were analysed using One-Way ANOVA followed by Tukey’s post hoc test for multiple comparisons among groups. Real-Time PCR data were analysed using Two-Way ANOVA followed by Bonferroni post hoc correction to account for multiple comparisons between factors. Data were expressed as mean ± SD; probability was considered statistically significant at *p* < 0.05.

## 5. Conclusions

In conclusion, both Gd and Er are teratogenic to *Xenopus laevis* embryos. However, Er induces more severe dysregulation of genes involved in development, detoxification, and inflammation, resulting in a higher incidence of malformations. Both REEs are confirmed as hazardous environmental contaminants, and their discharge into surface waters should be better controlled.

## Figures and Tables

**Table 1:** Effects of Gadolinium and Erbium on *Xenopus laevis* embryo.

Gadolinium				
	Control	40 µg/L	60 µg/L	80 µg/L
Embryos (*n*)	90	90	90	90
Dead embryos (*n*)	13	19	20	18
Living embryos (*n*)	77	71	70	72
Mortality (%)	14.4	21.1	22.2	20
Malformation (*n*, %)	2 (2.6)	6 (8.4) ^a^	6 (8.4) ^a^	6 (8.2) ^a^
Erbium				
	Control	1 µg/L	10 µg/L	20 µg/L
Embryos (*n*)	90	90	90	90
Dead embryos (*n*)	13	13	11	13
Living embryos (*n*)	77	77	79	77
Mortality (%)	14.4	14.4	12.2	14.4
Malformation (*n*, %)	1 (1.2)	4 (5.2)	7 (8.86) ^a^	10 (13) ^b,c^

Mortality and malformation rates were analysed using the Chi-square (χ^2^) test. Asterisks indicate significant differences compared with the control group (^a^ *p* < 0.05; ^b^ *p* < 0.01; Chi-square (χ^2^) test for trend ^c^ *p* < 0.001). Values are expressed as the number of embryos (*n*) and the percentage (%).

## Data Availability

The original contributions presented in this study are included in the article/Supplementary Material. Further inquiries can be directed to the corresponding authors.

## Supplementary Materials

The following supporting information can be downloaded at: https://www.mdpi.com/article/10.3390/ijms27072920/s1.

## References

[1] Yan S., Xu S., Lei S., Gao Y., Chen K., Shi X., Guo Y., Bilyera N., Yuan M., Yao H. (2024). Hyperaccumulator extracts promoting the phytoremediation of rare earth elements (REEs) by *Phytolacca americana*: Role of active microbial community in rhizosphere hotspots. Environ. Res..

[2] Guo Y., Chen K., Lei S., Gao Y., Yan S., Yuan M. (2023). Rare Earth Elements (REEs) adsorption and detoxification mechanisms in cell wall polysaccharides of *Phytolacca americana* L.. Plants.

[3] Balaram V. (2019). Rare earth elements: A review of applications, occurrence, exploration, analysis, recycling, and environmental impact. Geosci. Front..

[4] MacMillan G.A., Chételat J., Heath J.P., Mickpegak R., Amyot M. (2017). Rare earth elements in freshwater, marine, and terrestrial ecosystems in the eastern Canadian Arctic. Environ. Sci. Process. Impacts.

[5] Ribeiro P.G., de Oliveira C., Guerra M.B.B., de Carvalho T.S., Martins G.C., da Silveira Pereira W.V., Ramos S.J., Guilherme L.R.G. (2024). Rare earths as emerging trace-element contaminants in soil. Curr. Pollut. Rep..

[6] Parant M., Perrat E., Wagner P., Rosin C., Py J.S., Cossu-Leguille C. (2018). Variations of anthropogenic gadolinium in rivers close to wastewater treatment plant discharges. Environ. Sci. Pollut. Res..

[7] Chen Z., Li Z., Chen J., Kallem P., Banat F., Qiu H. (2022). Recent advances in selective separation technologies of rare earth elements: A review. J. Environ. Chem. Eng..

[8] Gwenzi W., Mangori L., Danha C., Chaukura N., Dunjana N., Sanganyado E. (2018). Sources, behaviour, and environmental and human health risks of high-technology rare earth elements as emerging contaminants. Sci. Total Environ..

[9] Malhotra N., Hsu H.S., Liang S.T., Roldan M.J.M., Lee J.S., Ger T.R., Hsiao C.D. (2020). An updated review of the toxicity effects of the rare earth elements (REEs) on aquatic organisms. Animals.

[10] Tao Y., Shen L., Feng C., Yang R., Qu J., Ju H., Zhang Y. (2022). Distribution of rare earth elements (REEs) and their roles in plant growth: A review. Environ. Pollut..

[11] Wang L., Han X., Ding S., Liang T., Zhang Y., Xiao J., Dong L., Zhang H. (2019). Combining multiple methods for provenance discrimination based on rare earth element geochemistry in lake sediment. Sci. Total Environ..

[12] Arienzo M., Ferrara L., Trifuoggi M., Toscanesi M. (2022). Advances in the fate of rare earth elements (REE) in transitional environments: Coasts and estuaries. Water.

[13] Merschel G., Bau M. (2015). Rare earth elements in the aragonitic shell of freshwater mussel *Corbicula fluminea* and the bioavailability of anthropogenic lanthanum, samarium and gadolinium in river water. Sci. Total Environ..

[14] Martino C., Chianese T., Chiarelli R., Roccheri M.C., Scudiero R. (2022). Toxicological impact of rare earth elements (REEs) on the reproduction and development of aquatic organisms using sea urchins as biological models. Int. J. Mol. Sci..

[15] Rogowska J., Olkowska E., Ratajczyk W., Wolska L. (2018). Gadolinium as a new emerging contaminant of aquatic environments. Environ. Toxicol. Chem..

[16] Trapasso G., Chiesa S., Freitas R., Pereira E. (2021). What do we know about the ecotoxicological implications of the rare earth element gadolinium in aquatic ecosystems?. Sci. Total Environ..

[17] Tornero A.M.V., Hanke G. (2017). Potential Chemical Contaminants in the Marine Environment: An Overview of Main Contaminant Lists.

[18] Pagano G. (2016). Rare Earth Elements in Human and Environmental Health: At the Crossroads Between Toxicity and Safety.

[19] Galdiero E., Carotenuto R., Siciliano A., Libralato G., Race M., Lofrano G., Fabbricino M., Guida M. (2019). Cerium and erbium effects on *Daphnia magna* generations: A multiple endpoints approach. Environ. Pollut..

[20] Oral R., Bustamante P., Warnau M., D’Ambra A., Guida M., Pagano G. (2010). Cytogenetic and developmental toxicity of cerium and lanthanum to sea urchin embryos. Chemosphere.

[21] Pagano G., Guida M., Tommasi F., Oral R. (2015). Health effects and toxicity mechanisms of rare earth elements: Knowledge gaps and research prospects. Ecotoxicol. Environ. Saf..

[22] Carotenuto R., Pallotta M.M., Tussellino M., Fogliano C. (2023). *Xenopus laevis* as a model organism for bioscience: A historic review and perspective. Biology.

[23] Mouche I., Malésic L., Gillardeaux O. (2017). FETAX assay for evaluation of developmental toxicity. Methods Mol. Biol..

[24] (2019). Standard Guide for Conducting the Frog Embryo Teratogenesis Assay-Xenopus (FETAX).

[25] Bezerra C.W.F., Palacio-Cortés A.M., Dos Santos M.P., Alves A.C.F., Schafaschek A.M., Grassi M.T., Navarro-Silva M.A. (2025). Biochemical and molecular effects of gadolinium and lanthanum on *Chironomus sancticaroli*. Arch. Environ. Contam. Toxicol..

[26] Haley T.J., Koste L., Komesu N., Efros M., Upham H.C. (1966). Pharmacology and toxicology of dysprosium, holmium, and erbium chlorides. Toxicol. Appl. Pharmacol..

[27] Mair A.R., Woolley J., Martinez M. (2010). Cardiovascular effects of intravenous gadolinium administration to anaesthetised dogs undergoing magnetic resonance imaging. Vet. Anaesth. Analg..

[28] Groarke J.D., Waller A.H., Vita T.S., Michaud G.F., Di Carli M.F., Blankstein R., Kwong R.Y., Steigner M. (2014). Feasibility study of electrocardiographic- and respiratory-gated, gadolinium-enhanced magnetic resonance angiography of the pulmonary veins and the impact of heart rate and rhythm on study quality. J. Cardiovasc. Magn. Reson..

[29] Li G.R., Baumgarten C.M. (2001). Modulation of cardiac Na^+^ current by gadolinium, a blocker of stretch-induced arrhythmias. Am. J. Physiol. Heart Circ. Physiol..

[30] Nilsson M.F., Ritchie H., Webster W.S. (2013). The effect on rat embryonic heart rate of Na^+^, K^+^;, and Ca^2+^ channel blockers, and the human teratogen phenytoin, changes with gestational age. Birth Defects Res. B.

[31] Zhang H., Walcott G.P., Rogers J.M. (2018). Effects of gadolinium on cardiac mechanosensitivity in whole isolated swine hearts. Sci. Rep..

[32] Baykara M., Ozcan M., Bilgen M., Kelestimur H. (2021). Interference of gadolinium dechelated from MR contrast agents by calcium signaling in neuronal cells of GnRH. J. Cell. Physiol..

[33] Quinn T.A., Kohl P. (2021). Cardiac mechano-electric coupling: Acute effects of mechanical stimulation on heart rate and rhythm. Physiol. Rev..

[34] Zhao Y., Liang J., Meng H., Yin Y., Zhen H., Zheng X., Zhang K. (2020). Rare earth elements lanthanum and praseodymium adversely affect neural and cardiovascular development in zebrafish (*Danio rerio*). Environ. Sci. Technol..

[35] Incardona J.P., Scholz N.L. (2016). The influence of heart developmental anatomy on cardiotoxicity-based adverse outcome pathways in fish. Aquat. Toxicol..

[36] Breckenridge R.A., Mohun T.J., Amaya E. (2001). A role for BMP signalling in heart looping morphogenesis in Xenopus. Dev. Biol..

[37] Burggren W.W., Pinder A.W. (1991). Ontogeny of cardiovascular and respiratory physiology in lower vertebrates. Annu. Rev. Physiol..

[38] Pan X., Li J., He X., Deng J., Dong F., Wang K., Yu S. (2019). Gadolinium chloride promotes proliferation of HEK293 human embryonic kidney cells by activating EGFR/PI3K/Akt and MAPK pathways. Biometals.

[39] Oral R., Pagano G., Siciliano A., Gravina M., Palumbo A., Castellano I., Migliaccio O., Thomas P.J., Guida M., Tommasi F. (2017). Heavy rare earth elements affect early life stages in *Paracentrotus lividus* and *Arbacia lixula* sea urchins. Environ. Res..

[40] Mohamed H.R., Elberry Y.A., Magdy H., Ismail M., Michael M., Eltayeb N., Safwat G. (2025). Erbium oxide nanoparticles induce potent cell death, genomic instability and ROS-mitochondrial dysfunction-mediated apoptosis in U937 lymphoma cells. Naunyn-Schmiedeb. Arch. Pharmacol..

[41] Li M., Yuan W., Duan S., Li Y., Zhang S., Zhao Y., Xiao S., Zhong K. (2025). The rare earth element erbium induces immune toxicity via the ROS/NF-κB pathway in zebrafish. Fish Shellfish Immunol..

[42] Fogliano C., Motta C.M., Venditti P., Fasciolo G., Napolitano G., Avallone B., Carotenuto R. (2022). Environmental concentrations of a delorazepam-based drug impact on embryonic development of non-target *Xenopus laevis*. Aquat. Toxicol..

[43] Park J., Hong T., An G., Park H., Song G., Lim W. (2023). Triadimenol promotes the production of reactive oxygen species and apoptosis with cardiotoxicity and developmental abnormalities in zebrafish. Sci. Total Environ..

[44] Keane J.A., Ealy A.D. (2024). An overview of reactive oxygen species damage occurring during in vitro bovine oocyte and embryo development and the efficacy of antioxidant use to limit these adverse effects. Animals.

[45] Biandolino F., Prato E., Grattagliano A., Libralato G., Trifuoggi M., Parlapiano I. (2024). Potential toxicity of nine rare earth elements (REEs) on marine copepod *Tigriopus fulvus*. J. Xenobiotics.

[46] Fogliano C., Di Marino S., Avallone B., Trifuoggi M., Siciliano A., Galdiero E., Chianese E., Mastrantone R., Pacchini S., Piva E. (2025). Developmental toxicity of co-exposure of heavy metal and polystyrene microplastics in *Xenopus laevis* embryo. Sci. Total Environ..

[47] Park M.J., Chae J.P., Woo D., Kim J.Y., Bae Y.C., Lee J.Y., Lee S.Y., Nam E.J., Nam S.W. (2024). Ibuprofen-induced multiorgan malformation during embryogenesis in *Xenopus laevis* (FETAX). Biochem. Biophys. Res. Commun..

[48] Grzymkowski J.K., Chiu Y.C., Jima D.D., Wyatt B.H., Jayachandran S., Stutts W.L., Nascone-Yoder N.M. (2024). Developmental regulation of cellular metabolism is required for intestinal elongation and rotation. Development.

[49] Bartlett H.L., Weeks D.L. (2008). Lessons from the lily pad: Using Xenopus to understand heart disease. Drug Discov. Today Dis. Models.

[50] Carotenuto R., Fogliano C., Rienzi M., Siciliano A., Salvatore M.M., De Tommaso G., Guida M. (2021). Comparative toxicological evaluation of tattoo inks on two model organisms. Biology.

[51] Navarro A., Weißbach S., Faria M., Barata C., Pina B., Luckenbach T. (2012). Abcb and Abcc transporter homologs are expressed and active in larvae and adults of zebra mussel and induced by chemical stress. Aquat. Toxicol..

[52] Bošnjak I., Lepen Pleić I., Borra M., Mladineo I. (2013). Quantification and in situ localisation of abcb1 and abcc9 genes in toxicant-exposed sea urchin embryos. Environ. Sci. Pollut. Res..

[53] Sawicki W.T., Kujawa M., Jankowska-Steifer E., Mystkowska E.T., Hyc A., Kowalewski C. (2006). Temporal/spatial expression and efflux activity of ABC transporter, P-glycoprotein/Abcb1 isoforms and Bcrp/Abcg2 during early murine development. Gene Expr. Patterns.

[54] Armstrong N.J., Fagotto F., Prothmann C., Rupp R.A. (2012). Maternal Wnt/β-catenin signaling coactivates transcription through NF-κB binding sites during Xenopus axis formation. PLoS ONE.

[55] Richardson J.C., Gatherer D., Woodland H.R. (1995). Developmental effects of over-expression of normal and mutated forms of a Xenopus NF-κB homologue. Mech. Dev..

[56] Singh A.K., Fechtner S., Chourasia M., Sicalo J., Ahmed S. (2019). Critical role of IL-1α in IL-1β–induced inflammatory responses: Cooperation with NF-κBp65 in transcriptional regulation. FASEB J..

[57] Nieuwkoop P.D., Faber J. (1956). Normal Table of Xenopus laevis (Daudin): A Systematical and Chronological Survey of the Development from the Fertilized Egg Till the End of Metamorphosis.

[58] Carotenuto R., Tussellino M., Ronca R., Benvenuto G., Fogliano C., Fusco S., Netti P.A. (2022). Toxic effects of SiO_2_NPs in early embryogenesis of *Xenopus laevis*. Chemosphere.

[59] Livak K.J., Schmittgen T.D. (2001). Analysis of relative gene expression data using real-time quantitative PCR and the 2^−ΔΔCT^ method. Methods.

[60] Abdi H., Williams L.J. (2010). Principal component analysis. WIREs Comput. Stat..

