# Supplementary material for: Impact of Erbium and Gadolinium on Xenopus laevis Embryo Development: A Study of Rare Earth Element Toxicity
Source: Int J Mol Sci. 2026 Mar 24;27(7):2920. doi: 10.3390/ijms27072920 (PMC13073799; doi:10.3390/ijms27072920)
Supplement: Supplementary file 1 [file ijms-27-02920-s001.zip › ijms-4197483-supplementary/ijms-4197483-supplementary.pdf]

TABLE S1. Statistical values of gene expression related to Gadolinium treatment

**Gadolinium: differences in gene expression**		
**GENE**	**TREATMENT**	**P VALUE**
tnfa	40 µg/L<br>60 µg/L<br>80 µg/L	<0,0001 (****)<br><0,0001 (****)<br><0,0001 (****)
il1b	40 µg/L<br>60 µg/L<br>80 µg/L	<0,0001 (****)<br><0,0001 (****)<br><0,0001 (****)
p65	40 µg/L<br>60 µg/L<br>80 µg/L	0,0003 (***)<br><0,0001 (****)<br><0,0001 (****)
abcb1	40 µg/L<br>60 µg/L<br>80 µg/L	0,0035 (**)<br>0,0002 (***)<br><0,0001 (****)
bmp4	40 µg/L<br>60 µg/L<br>80 µg/L	0,0024 (**)<br>0,0003 (***)<br>0,1511 (ns)
fgf8	40 µg/L<br>60 µg/L<br>80 µg/L	0,3460 (ns)<br><0,0001 (****)<br><0,0001 (****)
egr2	40 µg/L<br>60 µg/L<br>80 µg/L	<0,0001 (****)<br><0,0001 (****)<br>0,4202 (ns)
sox9	40 µg/L<br>60 µg/L<br>80 µg/L	0,0006 (***)<br>0,0011 (**)<br><0,0001 (****)
pax6	40 µg/L<br>60 µg/L<br>80 µg/L	0,4802 (ns)<br>0,0216 (*)<br>0,0009 (***)
rax1	40 µg/L<br>60 µg/L<br>80 µg/L	0,0013 (**)<br><0,0001 (****)<br>0,0040 (**)

Table S2: Statistical values of gene expression related to Erbium treatment

**Erbium: differences in gene expression**		
**GENE**	**TREATMENT**	**P VALUE**
tnfa	1 µg/L	>0,9999 (ns)
	10 µg/L	<0,0001 (****)
	20 µg/L	<0,0001 (****)
il1b	1 µg/L	<0,0001 (****)
	10 µg/L	<0,0001 (****)
	20 µg/L	<0,0001 (****)
p65	1 µg/L	0,0003 (***)
	10 µg/L	<0,0001 (****)
	20 µg/L	<0,0001 (****)
abcb1	1 µg/L	0,0024 (**)
	10 µg/L	<0,0001 (****)
	20 µg/L	<0,0001 (****)
bmp4	1 µg/L	0,9995 (ns)
	10 µg/L	<0,0001 (****)
	20 µg/L	0,0275 (*)
fgf8	1 µg/L	0,6783 (ns)
	10 µg/L	0,6312 (ns)
	20 µg/L	0,2343 (ns)
egr2	1 µg/L	<0,0001 (****)
	10 µg/L	<0,0001 (****)
	20 µg/L	0,9909 (ns)
sox9	1 µg/L	0,2120 (ns)
	10 µg/L	0,0246 (*)
	20 µg/L	0,1701 (ns)
pax6	1 µg/L	0,9951 (ns)
	10 µg/L	0,9568 (ns)
	20 µg/L	<0,0001 (****)
rax1	1 µg/L	<0,0001 (****)
	10 µg/L	<0,0001 (****)
	20 µg/L	0,8417 (ns)
